# CAREGIVER ORAL HEALTH BELIEFS, CONVERSATIONS, AND ACCESS: FINDINGS FROM THE MOTIVATE AT HOME PROGRAM

**DOI:** 10.1093/geroni/igad104.0415

**Published:** 2023-12-21

**Authors:** Jennifer Crittenden, Denise O’Connell, Labrini Nelligan

**Affiliations:** University of Maine Center on Aging, Bangor, Maine, United States; Lunder-Dineen Health Education Alliance of Maine, Bangor, Maine, United States; Massachusetts General Hospital, Boston, Massachusetts, United States

## Abstract

The number of caregivers across the nation is growing. A three-way collaboration is examining their knowledge and attitudes related to the connection between oral health and overall health. Currently, little is known about caregiver oral health beliefs and how they relate to oral health care for older adult care recipients (CRs). Survey data from the MOTIVATE at Home program from 135 caregivers/care partners (CPs) across rural Maine were analyzed (age: M = 61 years, SD = 13, range= 25 to 85; 85.2% female). Care partners who felt they have a role in preventing tooth decay were more likely to report an oral health visit in the last year for their CR (M = .75, SD = .438; t(84)= -2.66, p=.009). Caregivers whose CR had not had dental care in the last year were less comfortable talking with dental providers (M = 1.50, SD = .632; t(92.84)= -3.01, p = .003), and non-dental health care providers (M = 1.53, SD =.754; t(94.06) = -2.42, p=.017) about oral health. For CRs who do not have a regular dentist, CPs were less comfortable in speaking with the CR about oral health care (M = 1.58, SD = .751; t(42.13) = -2.23; p=.031). These findings are currently informing the development of an educational intervention for CRs which will: Emphasize the importance of regular oral health visits; Improve understanding of the CP role in promoting oral health; Provide tools for conversations with a range of providers.

